# 24-hour access outpatient clinic for patients with exacerbation of chronic disease: a before-after cohort study of differences in acute healthcare utilisation

**DOI:** 10.1186/s12913-018-3475-1

**Published:** 2018-08-28

**Authors:** Anders Damgaard Møller, David Høyrup Christiansen, Cathrine Bell, Ulrich Fredberg, Peter Vedsted

**Affiliations:** 1 Diagnostic Centre, University Research Clinic for Innovative Patient Pathways, Silkeborg Regional Hospital, Department of Clinical Medicine, Aarhus University, Silkeborg, Denmark; 2 Department of Occupational Medicine, Regional Hospital West Jutland – University Research Clinic, Aarhus, Denmark; 3 Department of Clinical Medicine, HEALTH, Aarhus University, Aarhus, Denmark; 4 Research Unit for General Practice, Department of Public Health, Aarhus University, Aarhus, Denmark

**Keywords:** Chronic disease, Integrated healthcare systems, Ambulatory care, Delivery of healthcare, Before-after study, Hotlines

## Abstract

**Background:**

Chronic diseases are becoming more common due to an increasing ageing population. Patients with chronic conditions managed in outpatient clinics account for a large share of healthcare costs. We developed a 24-h access outpatient clinic offering 24-h telephone support and triaged access to the hospital for patients with acute exacerbation of four selected chronic diseases. The aim of this study was to conduct a 1-year before-after study of the acute healthcare utilisation in patients offered the 24-h access outpatient clinic intervention.

**Methods:**

The study was conducted as an observational register-based cohort study. Data from the patient administrative register and the Danish National Health Service Register were extracted 12 months before and 12 months after implementation of the 24-h access intervention. Patients with chronic obstructive pulmonary disease, chronic liver disease, inflammatory bowel disease and heart failure managed in hospital outpatient clinics were enrolled in the study. Differences in healthcare utilisation were analysed for all patients, including the subgroup of high-risk patients with at least one acute admission in the year before enrolment.

**Results:**

Length-of-stay remained unchanged for all diagnostic groups, except for patients with heart failure in whom a statistically significant reduction was observed. Statistically significant reductions of length of stay and acute admissions were observed in all high-risk groups, except for patients with chronic liver disease. A statistically significant reduction in the number of contacts to out-of-hours primary care was seen in patients with chronic obstructive pulmonary disease, whereas the level remained unchanged in the other diagnostic groups. Similar patterns were also seen in high-risk patients.

**Conclusions:**

The 24-h access outpatient clinic did not increase the use of acute healthcare services inpatients with chronic disease. Significant reductions in hospital utilisation were seen in high-risk patients. These preliminary results should be interpreted with caution due to the observational before-after design of the study.

## Background

Better living conditions and medical advances have extended the average life expectancy in western countries [1]. This has increased the number of individuals living with chronic disease, and evidence suggests that the proportion will rise in the future [2]. As a consequence, healthcare expenditures, and the ability to deliver high quality healthcare, are inevitably threatened by the increased demand.

In Denmark the majority of patients suffering from chronic disease are managed by their general practitioner (GP) and only rarely need assistance from the secondary healthcare sector [3]. Patients characterised by a more advanced level of chronic disease with a need for specialised care are, in contrast, managed in hospital outpatient clinics [3]. This latter group is particularly vulnerable in the increasingly fragmented healthcare system [4]. These patients more often have exacerbations and are frequently in contact with the healthcare system. This situation calls for a more proactive and tailored approach [5] applying innovative solutions in healthcare delivery that fulfil the triple aim of improving health outcomes and the experience of care while reducing costs [6]. Such new initiatives could provide better use of healthcare resources and possibly higher quality of services.

Integrated healthcare has been suggested as a core component for optimising healthcare delivery for patients with multidisciplinary and complex needs. Although the notion of integrated healthcare is inconsistently defined, there is general agreement that it requires coordination of care within and across healthcare-providing institutions [7]. A recent umbrella review reported positive trends in favour of integrated care interventions as these interventions tend to reduce healthcare utilisation in patients with chronic disease. [8]. Earlier studies have found that patients with chronic disease generally emphasise that integrated healthcare systems should be highly accessible and adaptable to their individual needs [9, 10].

The Regional Hospital Silkeborg in Denmark has developed and implemented the concept of a 24-h access outpatient clinic. This outpatient clinic offers 24-h telephone access and, if necessary, subsequent examination and treatment for patients with acute exacerbation of specific chronic diseases.

The aim of this 1-year before-after study was to investigate the healthcare utilisation in terms of acute hospital care and general practice services in a cohort of patients who were offered the 24-h access outpatient clinic intervention. We hypothesised that no significant changes between the before and after periods in hospital hourly length of stay (LOS), number of acute admissions and number of contacts to GP out-of-hours services would be observed. However, we expected that the subgroup of patients with at least one acute admission during the year before study enrolment would benefit from the intervention and show significant reductions in the same outcome measures.

## Methods

### Design

The study was conducted as a cohort study with a before and after analysis. Included patients were followed during 12 months before and 12 months after being offered triaged access to a 24-h outpatient clinic.

### Setting

The 24-h outpatient clinic was established at the Diagnostic Centre, Silkeborg Regional Hospital, Denmark. The hospital has a catchment area of approx. 177,000 citizens aged 18 years or older [11]. The hospital is located in the Central Denmark Region, one of five Danish regions managing the healthcare services for approx. 1.3 million of the approx. 5.8 million Danish citizens [12]. Healthcare in Denmark is based on a tax-funded public system that provides universal and free coverage for all Danish citizens [13].

The responsibility for the Danish healthcare system is divided between the national state, five regions and 98 municipalities. The national state provides the legal framework and coordinates healthcare delivery in the regions and municipalities. Key regional tasks are controlling public hospitals and financing the private GP’s and practicing specialists. The municipalities are mainly concerned with local nursing homes, home care, disease prevention and health promotion activities [13].

The entry point to the acute healthcare services is the GP, who acts as a gatekeeper to other healthcare services [14]. The GPs run out-of-hours large-scale cooperatives with central triage from 4 pm to 8 am on weekdays and during all weekends and holidays. Since 2007, Danish hospitals have worked towards a single-entry system through emergency departments (ED) for all patients referred for acute admission [15]. This process has included a reduction and centralisation of hospitals offering services for patients with acute needs.

### Participants

Inclusion criteria for enrolment in the 24-h outpatient clinic were: 1) residence in Silkeborg Municipality, 2) contact with an outpatient clinic at Silkeborg Regional Hospital in the study period and 3) primary diagnosis of at least one of four selected chronic diseases: inflammatory bowel disease (IBD) (International Classification of Disease, 10th revision (ICD-10): DK50-DK52), chronic liver disease (ICD-10: DK658I, DK702-DK704, DK711, DK717, DK72-DK74, DK754, DK761, DK766-DK767, DI85), chronic obstructive pulmonary disease (COPD) (ICD-10: DJ44.0,DJ44.1 or DJ44.9) and heart failure (ICD-10: DI11.0, DI13.0, DI13.2, DI42.0, DI42.6-DI42.9, DI50.0- DI50.1 or DI50.9). The selection of these particular diagnoses was based on specialist clinician assessments of which chronic diseases covered by the hospital would benefit from the intervention.

The 24-h outpatient clinic was implemented in the last three months of 2015. All patients fulfilling the inclusion criteria received a short information letter on their possibility to call the outpatient clinic at any time when experiencing exacerbation symptoms related to the qualifying chronic disease. Subsequent incident cases of patients fulfilling the inclusion criteria were consecutively offered access to the 24-h outpatient clinic. This study is based on the cohort of prevalent patients enrolled at implementation.

### Intervention

**Fig. 1:**
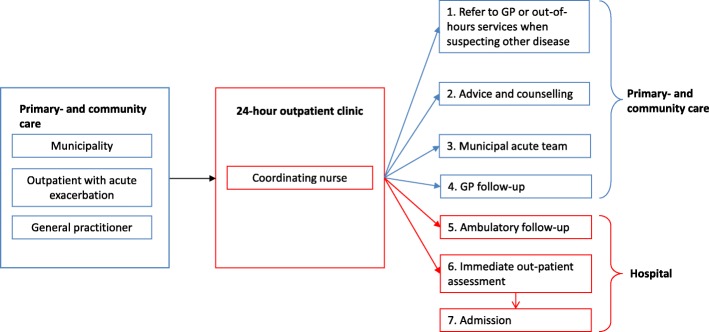
Patient flow for patients with acute exacerbation enrolled in the 24-h outpatient clinic. GP; general practitioner

Patients and their relatives, the GP and the home nurse could independently call the 24-h outpatient clinic. Specialised nurses handled the calls to provide qualified help at the lowest cost (Fig. 1). The specialised triage nurses were instructed to seek solutions in the patient’s immediate environment (e.g. municipal acute nurse services or GP). Upon indication, the patient received immediate assessment in an outpatient setting at the hospital. If the situation could not be managed here, the patient was hospitalised.

The 24-h outpatient clinic intervention differentiates from usual care by providing round-the-clock access to the hospital at different levels of care and allowing the patient to bypass the GP(daytime and out-of-hours services) in situations related to exacerbation of the qualifying chronic disease. Moreover, the 24-h access patient pathway differs because it omits the emergency department (ED) as the entry point for acute hospital admission.

### Outcome measures

The outcome measures were hourly LOS per year, number of acute hospital admissions and number of contacts to GP out-of-hours services per year. Data on hospital utilisation was based on all-cause acute admissions. Data was obtained from the hospital patient administration system (PAS), which contains data on patient contacts. The content in this system is comparable to the highly complete Danish National Patient Register, which is known for high validity [16]. Data on contacts with GPs was collected from the Danish National Health Service Register [17]. Complete data linkage was possible through the unique personal identification number assigned to all Danish citizens [18]. Information on vital status (death or immigration) was collected from the PAS. Only participants with complete 12-month follow-up were included in the analyses.

### Statistical analysis

Interval data was reported by mean and standard deviation (SD) for normally distributed results, whereas median and interquartile interval (IQI) was used for non-normally distributed results. Utilisation data was treated as count data and reported by median and IQI.

Differences between before and after results were tested for statistical significance with the Wilcoxon signed-rank test for all patients and for the subgroup defined as high-risk patients with at least one acute admission in the before period. We expected the data on healthcare utilisation to be characterised by a zero-inflated right skewed distribution. By restricting a subgroup analysis to patients with utilisation in the before period, we took into account that patients with zero values in the before period had no potential for reductions in utilisation. The data on utilisation was reported by totals in the before and after analyses.

Proportions of patients with at least one acute admission in the before- and after period were calculated. Odds ratios of experiencing at least one acute admission in the after period compared to the before period was estimated in a generalised estimating equations (GEE) model which is appropriate for repeated or clustered data. We fitted an unadjusted GEE model and an adjusted model including the variables sex and age. These analyses included all patients.

The significance level was set at 5% for all tests. All data was analysed using Stata, version 15 (Stata Corporation, College Station, Texas).

## Results

In total, 822 patients were offered access to the 24-h outpatient clinic. Of these, 53 (6%) died and 3 (0.4%) relocated outside Denmark during the follow-up period. In total, 766 patients were included in the analyses. The mean age was highest for patients with COPD (mean = 68 years, SD = 12) and lowest for patients with IBD (mean = 49 years, SD = 16) (Table 1).

**Table 1:** Characteristics of patients enrolled in the 24-h outpatient clinic with complete 12- month follow-up

	Heart failure (*n* = 130)	COPD (*n* = 154)	IBD (*n* = 437)	Chronic liver disease (*n* = 45)
Sex				
Female	37 (28%)	80 (52%)	242 (55%)	23 (51%)
Male	93 (72%)	74 (48%)	195 (45%)	22 (49%)
Age (years)				
18–40	5 (4%)	1 (1%)	143 (33%)	0 (0%)
41–60	28 (22%)	30 (19%)	173 (40%)	24 (53%)
61–75	60 (46%)	74 (48%)	100 (23%)	21 (47%)
> 75	37 (28%)	49 (32%)	21 (5%)	0 (0%)
Mean (SD)	68 (12)	69 (10)	49 (16)	60 (7)
Acute hospital admissions 12 months before enrolment				
0	71 (55%)	92 (60%)	374 (86%)	28 (62%)
1	31 (24%)	34 (22%)	41 (9%)	7 (16%)
2	6 (5%)	12 (8%)	12 (3%)	4 (9%)
3	12 (9%)	8 (5%)	7 (2%)	3 (7%)
> 3	10 (8%)	8 (5%)	3 (1%)	3 (7%)
Median (IQI)	0 (0; 1)	0 (0; 1)	0 (0; 0)	0 (0; 1)
GP daytime visits 12 months before enrolment				
0–5	33 (25%)	28 (18%)	224 (51%)	14 (31%)
6–10	28 (22%)	35 (23%)	117 (27%)	11 (24%)
11–20	44 (34%)	50 (33%)	63 (14%)	12 (27%)
21–30	18 (14%)	25 (16%)	23 (5%)	6 (13%)
> 30	7 (5%)	16 (10%)	10 (2%)	2 (4%)
Median (IQI)	11.5 (5; 19)	12 (7; 21)	5 (2; 10)	9 (4; 16)
GP out-of-hours services contacts 12 months before enrolment				
0	73 (56%)	65 (42%)	244 (56%)	20 (44%)
1	33 (25%)	37 (24%)	105 (24%)	10 (22%)
2	14 (11%)	17 (11%)	39 (9%)	8 (18%)
3	5 (4%)	16 (10%)	21 (5%)	2 (4%)
> 3	5 (4%)	19 (12%)	28 (6%)	5 (11%)
Median (IQI)	1 (1;2)	2 (1;3)	1 (1;2)	2 (1;3)

*Numbers (%) unless stated otherwise*. Percentages may not add up to 100 due to rounding. *COPD* chronic obstructive pulmonary disease, *IBD* inflammatory bowel disease, *SD* standard deviation, *IQI* interquartile interval, *GP* general practitioner

**Fig. 2:**
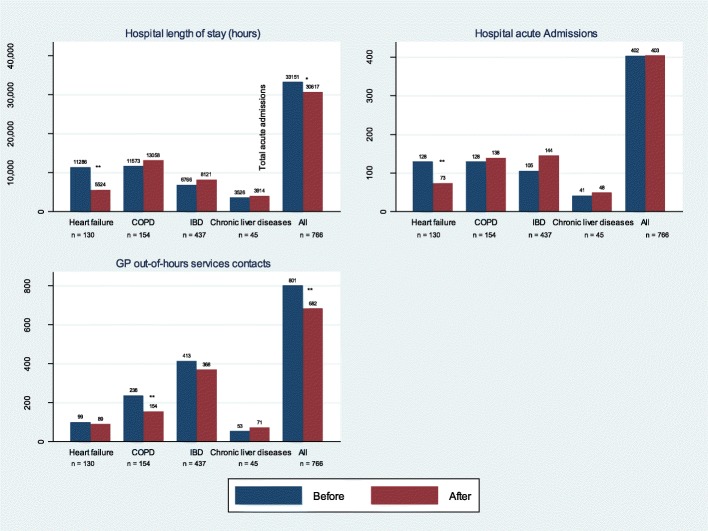
Healthcare utilisation 12 months before-after enrolment in the 24-h outpatient clinic – all patients. COPD; chronic obstructive pulmonary disease. IBD; inflammatory bowel disease. * *p-* value < .05. ** *p-* value < .01

**Fig. 3:**
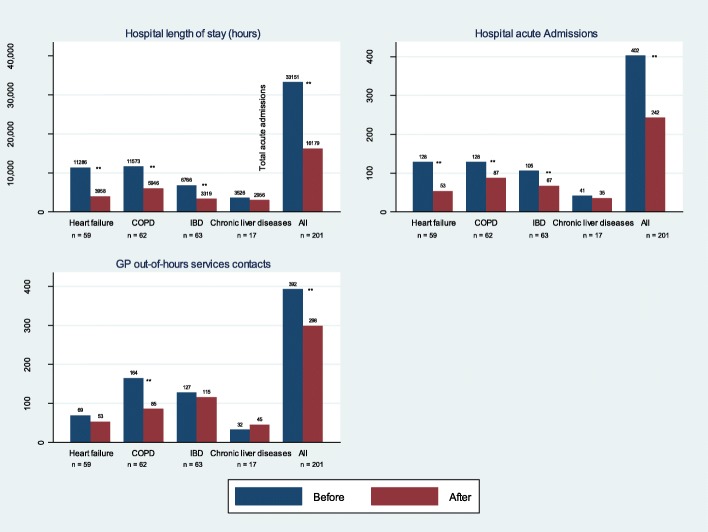
Healthcare utilisation 12 months before-after enrolment in the 24-h outpatient clinic – high-risk patients. COPD; chronic obstructive pulmonary disease. IBD; inflammatory bowel disease. ** *p-* value < .01

Total healthcare utilisation before and after implementation of the 24-h outpatient clinic is presented for all patients in Fig. 2 and for the subgroup of high-risk patients in Fig. 3.

### Length of stay

A statistically significant decrease in total LOS (*p* < .001) was observed for patients with heart failure, whereas minor increases were observed for patients with the other chronic diseases (Fig. 2). For the subgroup of high-risk patients, all diagnostic groups showed a decrease in LOS (Fig. 3). The reductions were statistically significant for all groups, except for the group of patients with chronic liver disease (*p* = .136).

### Acute admission

A statistically significant decrease in acute admissions was observed for patients with heart failure, and minor increases were observed for patients with the other chronic diseases (Fig. 2). The subgroup ofhigh-risk patients showed a decrease in acute admissions (Fig. 3); this decrease was statistically significant for all groups, except for patients with chronic liver disease (*p* = .328).

Proportions of patients experiencing at least one acute admission in the before- and after period are shown in Table 2. Except for patients with IBD (adjusted OR = 1.10, 95% CI [0.80, 1.50]), all groups showed lower propensity to be admitted after the introduction of the 24-h outpatient clinic; this was statistically significant for patients with heart failure (adjusted OR = 0.35, 95% CI [0.22, 0.55]).

**Table 2:** Proportions and odds ratios of admission 12 months before-after enrolment in the 24-h outpatient clinic

	Heart failure (*n* = 130)	COPD (*n* = 154)	IBD (*n* = 437)	Chronic liver disease (*n* = 45)	All (*n* = 766)
Before (95% CI)	45% (37, 54)	40% (32, 48)	14% (11, 18)	38% (24, 53)	26% (23, 30)
After (95% CI)	22% (15, 30)	38% (30, 46)	16% (12, 19)	29% (16, 44)	22% (19, 25)
Unadjusted OR (95% CI)	0.35 (0.22, 0.55	0.90 (0.59, 1.37)	1.09 (0.80, 1.50)	0.67 (0.34, 1.33)	0.79 (0.65, 0.96)
Adjusted OR (95% CI)	0.35 (0.22, 0.55)	0.90 (0.58, 1.37)	1.10 (0.80, 1.50)	0.63 (0.29, 1.38)	0.79 (0.64, 0.96)

*COPD* chronic obstructive pulmonary disease, *IBD* inflammatory bowel disease

### Contacts to GP out-of-hours services

Except for patients with chronic liver disease, all groups showed decreases in the number of contacts to GP out-of-hours services (Fig. 2). This was statistically significant for patients with COPD (*p < .001).* A similar pattern was observed for the subgroup of high-risk patients (Fig. 3).

## Discussion

### Summary of key findings

Establishing an outpatient clinic with 24-h access for patients with four selected chronic diseases did not result in increased utilisation of acute healthcare in these groups. Rather, in patients with heart failure, we observed statistically significant reductions in LOS and number of acute admissions. Likewise, the number of contacts to out-of-hours primary care was reduced in patients with COPD. All other measures remained unchanged. In high-risk patients, we observed statistically significant reductions in LOS, number of acute admissions and number of contacts to GP out-of-hours services. The largest reductions were observed in patients with heart failure, whereas patients with chronic liver disease did not show any statistically significant changes when measured on these outcomes.

### Strengths and limitations

The present study was based on a large sample of patients with complete registration and follow-up obtained from high quality registers [16–18]. A limitation of the study was that we were unable to discriminate between utilisation due to the specific chronic disease and other morbidities. Inevitably, healthcare utilisation caused by other reasons would be present in all included groups. As the study relied on register-based data, discrimination would be possible by specifying algorithms based on ICD-10 diagnoses to identify certain admission details. The sensitivity of diagnoses relevant to this study has been found acceptable, whereas little is known about the specificity [19].

Several studies have described the fallacies of interpreting results of observational before and after cohort studies as causal [20, 21]. Thus, the design of this study does not allow us to draw any conclusions about the effect of the intervention. The results could be explained as a natural development of a disease or regression towards the mean for utilisation. However, admission of patients with chronic disease is generally strongly predicted by prior admissions [22–24], which is inconsistent with the reductions observed in our study. Regression towards the mean would be a particular problem if patients with extreme healthcare utilisation before the intervention were included and then died or stopped using the healthcare services. Therefore, we excluded patients without complete follow-up. Furthermore, our statistical models were clearly limited since we were unable to adjust for changes in co-morbidity which could explain parts of the observed differences. Unfortunately, such measures are not adequately included in the PAS database. The occurrence of co-morbidity is characterised by a deterioration of health status. Therefore, the lack of inclusion has likely affected our results in the direction of underestimating the observed reductions and can not explain the observed results. It is however a particular weakness of the study that the information was not included in the statistical models and this should be considered carefully when interpreting the results.

A learning curve was expected for the triage nurses. Telephone triage constituted a new task, and it is likely that initial uncertainty may have contributed to conservative decisions. This could have favoured the choice of immediate assessment at the hospital, which is likely to have generated more acute admissions than necessary and to have underestimated the differences between the periods. Finally, some of the results are flawed by low statistical precision due to small groups.

### Comparison with other studies

Only few other studies have investigated 24-h telephone access to hospital services specifically targeting patients with chronic disease. Hurst et al. investigated the effects of a 24-h telephone support line (answered by a study nurse or physician) for COPD patients combined with a one-hour education session, instructions on when to call and bi-monthly follow-up calls for a one-year period and compared before and after results [25]. The population had a median of three (IQI: 1–4) exacerbations in the year before enrolment and was, therefore, comparable to the subgroup of high-risk patients in our study. They found a 45% reduction in admissions and a 37% reduction in annual LOS. In comparison, in our study, the relative reduction in admissions and in LOS was 49% and 32%, respectively.

Roberts et al. investigated a 24-h hotline intervention for patients with COPD and compared ED presentations between callers and non-callers in an observational study [26]. They found that callers had fewer presentations at the ED than non-callers. The authors highlighted that other factors, such as depression and anxiety, could contribute to ED presentations rather than just disease severity. Non-specialised staff is less aware of such needs, which may result in potentially avoidable acute admissions. A 24-h outpatient clinic is per definition always available and offers specialised knowledge about the disease, and the patient may thus use alternative strategies to acute admission.

Studies by Nightingale [27] and Thomson [28] have shown that providing helplines to specialist nurses for patients with IBD did not lead to increased clinical attendance. Their results are in line with ours as the availability of the 24-h access outpatient clinic did not cause overuse. In addition, Younge and Norton [29] stressed that helplines for IBD patients should not just be seen as a way of reducing contacts; it should rather be seen as an improvement that could contribute to better self-management of the disease.

### Implications

It could be hypothesised that open access to specialised hospital services would result in increased utilisation. We did not observe such trend, and previous studies have obtained similar results [25, 26]. Several explanations could be considered. For patients managed in outpatient clinics, the specialised clinic is the primary point of contact for matters concerning the chronic condition. Open access could provide the patient with a feeling of safety due to familiarity with the staff and round-the-clock access. The importance of accessibility has been demonstrated in earlier studies [9, 10]. When patients experience symptoms of exacerbation, it seems likely that a combination of easy access and the need for feeling safe could reduce the length of time from registration of symptom to decision about contacting the healthcare system and thereby permit early onset of treatment. Prompt action allows for treatment of patients with little resources and alternative strategies to acute admissions in the immediate environment assisted by the local health services or the GP.

Moreover, we saw a decreased use of GP out-of-hours services among COPD patients, which could indicate a more efficient pathway for this known exacerbation.

An additional implication could be establishment of a simplified healthcare system to provide better access for the patients and to promote empowerment as they are allowed to select the appropriate service to accommodate their current needs. The alternative in Denmark is a scenario with scheduled appointments in the outpatient clinic, contacts to GP (daytime and out-of-hours services) at exacerbations, acute admission to the ED and subsequent transfer to a specialised ward. This includes numerous handovers and fragmented care.

Future studies should investigate the 24-h outpatient clinic intervention in a controlled study design combined with analysis of cost-effectiveness. The patient perspective should also be investigated to provide an understanding of the personal implications.

## Conclusions

An intervention with a 24-h access outpatient clinic did not result in increased use of acute healthcare utilisation for patients with heart failure, COPD, IBD and chronic liver disease. High-risk patients with at least one acute admission in the year before enrolment showed reductions in LOS, in acute admissions and in GP out-of-hours services, except for patients with chronic liver disease. Due to the observational before-after design, the results of the study should be cautiously interpreted as cause and effect are generally hard to establish in this type of study design.

## Abbreviations

CI: Confidence interval
COPD: Chronic obstructive pulmonary disease
ED: Emergency department
GP: General practitioner
IBD: Inflammatory bowel disease
ICD: International classification of diseases
IQI: Interquartile interval
LOS: Length of stay
PAS: Patient administrative system
RR: Risk ratio
SD: Standard deviation
